# Enterocutaneous Fistula: A Simplified Clinical Approach

**DOI:** 10.7759/cureus.7789

**Published:** 2020-04-22

**Authors:** Faiz Tuma, Zachary Crespi, Christopher J Wolff, Drew T Daniel, Aussama K Nassar

**Affiliations:** 1 General Surgery, Central Michigan University College of Medicine, Saginaw, USA; 2 Surgery, Central Michigan University College of Medicine, Mount Pleasant, USA; 3 Surgery, Stanford University, Palo Alto, USA

**Keywords:** small bowel, fistula, malignancy, malnutrition, gastrointestinal, crohn's disease

## Abstract

A “fistula” is an abnormal connection between two epithelial surfaces. Fistulae are named based on the two surfaces or lumens they connect to. Fistulae form due to loss of wall integrity from an underlying insult, leading to the penetrance of an adjacent organ or epithelized surface. Common causes of small bowel fistulae include sequelae of surgical intervention, foreign body, bowel diverticula, Crohn's disease, malignancy, radiation, and infection. A histopathological analysis displays acute and/or chronic inflammation due to the underlying pathology.

A thorough history and physical examination are important components of patient evaluation. Generally, patients will present with non-specific constitutional symptoms in addition to local symptoms attributed to the fistula. In rare instances, symptoms may be severe and life-threatening. Initial laboratory workup includes complete blood count, comprehensive metabolic panel, and lactate level. Radiologic imaging is useful for definitive diagnosis and helps delineate anatomy. In practice, computed tomography (CT) is the initial imaging modality. The addition of intravenous or enteric contrast may be helpful in certain situations. Magnetic resonance imaging (MRI) may also be used in special circumstances. Invasive procedures, such as endoscopy, can assist in the evaluation of mucosal surfaces to diagnose pathology such as inflammatory processes.

Appropriate management should include optimizing nutritional status, delineating fistulous tract anatomy, skincare, and managing the underlying disease. A non-operative approach is generally accepted as the initial approach especially in the acute/subacute setting. However, operative intervention is indicated in the setting of failed non-operative management. Successful management of small bowel fistulae requires a multidisciplinary team approach.

To conclude, a small bowel fistula is a complex clinical disease, with surgical intervention being the most common cause in developed countries. The non-operative approach should be trialed before an operative approach is considered.

## Introduction and background

The term "fistula" refers to abnormal communication between two epithelial surfaces. This definition does not cover all the known types of fistulae. For example, an enteroatmospheric fistula is an example where the fistula connects the enteric epithelial lining to “air” or wound granulation tissue [1-3].

The classification of fistulae is based on two major criteria: anatomy and output. The anatomy of a fistula dictates its nomenclature and follows the two surfaces or lumens that are connected [4]. The name of the fistula usually starts from the origin of the fistula and ends in the connected adjacent organ. For example, an enterocutaneous fistula starts from the small bowel and ends in the skin [5]. Therefore, a small bowel fistula is a fistula that connects the small bowel to a variety of adjacent organs or surfaces. Common examples of intestinal fistulae are enterocutaneous fistula, entero-enteric fistula, enterovesical fistula, enterocolic fistula, enteroatmospheric fistula, and choledochoenteric fistula [6-8]. In addition, fistulae can be further classified depending on their 24-hour output to low output fistula (less than 200 ml/day), moderate output fistula (200-500 ml/day), and high output fistula (greater than 500 ml/day).

Fistulae are complex surgical conditions that pose a significant challenge to current surgical care [7]. Assessment, management, and prognosis depend on the underlying etiology, the complexity of the fistula, and the patients' comorbid factors [9-10]. Small bowel fistulae are, unfortunately, a common surgical disease of the gastrointestinal tract. Given that, small bowel fistulae will be the focus of discussion in this review article and other types of fistulae will not be discussed in this review.

## Review

Etiology

The etiologies of small bowel fistulae are as follows:

1. Surgical Complication

This is the most common cause of small bowel fistulae. Up to 80% of small bowel fistulae are reported to be postabdominal procedures [11]. Small bowel resection, Meckel's diverticulum resection, incisional hernia repair, adhesion-lysis, and drainage of intraabdominal collections are commonly reported in association with fistulae development. A fistula, in these settings, starts as an intraoperative injury to the small bowel. This subsequently causes the leakage of enteric contents, which may collect and form an intraabdominal pocket or abscess. Chronic inflammation in the area of the injury causes the breakdown of adjacent tissues and may eventually lead to the disruption of normal anatomic boundaries. This process is defined as a fistula when a new connection is created between two previously disconnected structures.

2. Bowel Diverticula

Diverticula, both false and true types, are a common cause of fistulae. Colonic diverticula fistulizing to the small bowel (coloenteric fistula) or small bowel diverticula (e.g. Meckel's, duodenal, or jejunal diverticula) connecting to other organs is relatively common [12]. It is thought that the perforation of the diverticulum (micro or macro), with subsequent acute inflammation and abscess formation, will erode and extend to an adjacent organ wall and create the fistulous connection. Occasionally, the pressure gradient on both ends of the fistula with the continued inflammatory process will likely maintain the fistula tract.

 *3. Crohn's Disease*

Chronic inflammatory bowel diseases, particularly Crohn's disease, is a well-known cause of small bowel fistula [13-15]. Entero-enteric, enterocolic, enterovesical, enterovaginal, and enterocutaneous fistulae are common examples of complications that occur in patients with Crohn's disease [7,16-17].

 *4. Malignancy*

Adenocarcinoma of the small bowel or adjacent organs is a known cause of fistulation to and from the small bowel [18]. These fistulae are also called malignant fistulae. Malignant tumors may spread radially. It is the pressure from this outward extension into other tissues that causes the breakdown of tissue planes and abnormal fistulous connections.

 5. Radiation

Radiation causes long-term chronic inflammation with poor healing and repair processes. Therefore, an intestinal fistula caused by radiation manifests after a long lag period that could extend several months or years and is particularly difficult to manage and heal [19].

 6. Non-surgical Injuries and Foreign Bodies

Injuries from trauma or by a foreign body can result in an abnormal fistulous connection due to a chronic granulomatous reaction [20].

7. Infectious

Infection is another potential etiology for fistulae, especially perianal fistulas. Common infections are tuberculosis and actinomycosis [21].

Once a fistula has formed, the long-term course is varied but many will spontaneously heal and close. The persistence of a fistula must make one suspicious for an ongoing pathology. These causes are outlined in the mnemonic "FRIEND": Foreign body, Radiation, Inflammation, Epithelization, Neoplasm, Distal obstruction. These pathologies will create an on-going milieu that promotes fistula persistence and must be investigated.

Epidemiology

The incidence of small bowel fistulae varies. An enterocutaneous fistula is the most common type and represents 88.2% of all fistulae [22]. Quinn M et al. reported, 89.1% of intestinal cutaneous fistula developed after abdominal surgery, followed by 6.88% occurring spontaneously, and 3.99% occurring after an endoscopic procedure [23]. In Crohn's disease, 21.7% of patients developed an enterocutaneous fistula on long-term follow-up [24]. Valle SJ et al. reported that 5.8% of patients developed an enterocutaneous fistula after cytoreductive surgery and hyperthermic intraperitoneal chemotherapy [25]. For an aortoenteric fistula, more than 75% involve the duodenum (aortoduodenal fistula) given its location from the graft [26]. One study reported that 0.81% of the endovascular aneurysm repair (EVAR) patients developed an aortoduodenal fistula [27]. A cholecystoduodenal fistula was a rare postcholecystectomy complication and presented in 0.42% of patients. A spontaneous cholecystoduodenal fistula due to chronic calculous cholecystitis could present as gallstone ileus causing 0.095% of mechanical small bowel obstruction [28].

Pathophysiology

As small bowel fistulae usually result from a complication of an underlying disease or injury, proper assessment and management depend on an understanding of the pathophysiology of the fistulae formation process [29]. The primary trigger of any fistula is a loss of bowel wall integrity due to an underlying insult. This will ultimately lead to leakage of bowel content, resulting in abscess formation and irritation and erosion to an adjacent organ or surface. The process may take from days to years depending on the underlying etiology. Iatrogenic surgical injuries may lead to intestinal fistulae within a few days while radiation may take from months to years.

Complex fistulae resulting from surgical procedures are formed by the leakage of intestinal contents that eventually find their way through the path of least resistance to another organ or surface and possibly erosions to more than a single organ. Iatrogenic controlled fistulae are intentionally formed for source control in the management of sepsis. An example of this is the acceptance of an ongoing bowel anastomotic leak controlled through an intraabdominally placed drain. The same concept applies to the external drainage of pancreatic fluid, which may accumulate after pancreatic surgery.

Histopathology

The histopathologic examination of an enterocutaneous fistula is usually performed postoperatively and is largely nonspecific. Findings usually show an acute-on-chronic inflammatory reaction in addition to the original pathology of the causative disease. The acute inflammation is caused by the primary pathology causing the fistula (diverticular disease, malignancy, etc.), tissue irritation by the flow of intestinal content, and the resulting infection [30]. Chronic inflammation is observed in radiation-induced fistula, Crohn's disease, malignancy, and chronic fistulae. Giant cell reaction is also seen in Crohn’s, tuberculosis, and actinomycosis. Identifying the fistula histopathology is usually done late and only after surgery. Occasionally, intraoperative diagnosis is made by completing a biopsy of incidentally identified fistulae. A frozen section is rarely used and is sometimes indicated to rule out malignancy to plan surgical excision. The surgical excision of malignancy involves en-bloc radical excision of the fistula and adjacent organs to achieve an R0 oncologic resection.

Postsurgical intestinal fistulae usually provoke an intense acute inflammatory reaction with a significant infectious bacterial translocation that may lead to sepsis. Severe septic shock with multiorgan system failure can be the presenting clinical picture on some of these occasions. Source control is the most crucial step in patients' survival in this scenario.

History and physical

The history and physical exam of patients with a small bowel fistula are an important component of evaluation and management planning. A variety of signs and symptoms can be identified in patients with these fistulae. On history, general non-specific constitutional symptoms are weakness, fever, chills, malaise, poor appetite, and protein and caloric malnutrition. Other specific symptoms are feculent/bilious wound discharge, diarrhea, and gastrointestinal (GI) bleed. Organ-specific symptoms are usually related to the organ involved in the fistula. Examples of organ-specific clinical manifestations are recurrent polymicrobial urinary tract infections (UTIs), pneumaturia, or fecaluria in a colovesical fistula [17]. Vaginal pain, feculent discharge, and recurrent infections are seen in a recto- or colovaginal fistula [16]. Skin pain, irritation, and excoriation are also seen in an entero or colocutaneous fistula. The more proximal the small bowel fistula, the worse the skin presentation, given the highly irritant secretions from the pancreas and stomach. In the acute phase of postsurgical small bowel fistula and leak, symptoms are usually more severe and can even be life-threatening given bacterial translocation from the raw, non-epithelialized surfaces. Sudden-onset deterioration of vital signs, severe abdominal pain, and peritoneal signs with tenderness could be a sign of an abscess or an intraperitoneal contamination/leak with concomitant sepsis.

Evaluation

The acuity of the fistula presentation will dictate the evaluation approach. Chronic or subacute fistulae, such as enterocutaneous, enterovesical, enterovaginal, or entero-enteric fistulae, are usually evaluated in an outpatient setting. The aim of the evaluation in this setting would be to (1) confirm the diagnosis, delineating anatomy with the characterization of the site, size, and complexity of the fistula, (2) identify the underlying disease, (3) plan for management, (4) re-evaluate, and (5) follow up progression. An acute small bowel fistula, as in a postsurgical complication, is usually evaluated in a hospital setting on an urgent basis to verify the diagnosis, rule out other complications, and evaluate appropriate sepsis workup and treatment.

Evaluation Modalities

Clinical: A clinical assessment usually starts with a thorough history and physical exam (see above).

Laboratory: 1. A complete blood count (CBC) to assess the white cell count, rule out blood loss anemia, and assess the hemoglobin level as compared to baseline. Additionally, a low MCV could indicate chronic blood loss anemia or malignancy; 2. A comprehensive metabolic panel, to assess electrolyte disturbances, kidney function, and hydration status; 3. Lactate, to assess tissue perfusion and guide resuscitation with other perfusion markers [31].

Imaging: Imaging with gastrointestinal (GI) contrast that traverses through the fistula (fistulogram) usually substantiates the diagnosis. On occasion, the contrast is not seen in the fistula itself but is seen in the end organ (bladder, vagina, extra-abdominally), which also provides the diagnosis [32]. Upper GI series, small bowel follow-through, or contrast enema can provide this confirmation. A computed tomography (CT) scan, however, is often the initial study, especially in an acute intestinal fistula. A CT scan is highly specific in delineating fistulous tract anatomy and often rules out the presence of an abdominopelvic abscess. A CT scan can also help with surgical planning. Magnetic resonance imaging (MRI) may also be necessary when the CT scan does not reveal a fistula but clinical suspicion remains. MRI has the advantage of better soft tissue characterization. It is also useful in complex fistulas such as in complicated Crohn’s disease [33]. A variant of MRI known as magnetic resonance enterorrhaphy is now widely used to rule out small bowel pathology and helps in delineating fistulous anatomy especially in Crohn’s disease [33].

Endoscopy: Colposcopy, cystoscopy, gastroduodenoscopy, bronchoscopy, and colonoscopy are occasionally utilized to aid the diagnosis of fistulas. This is done by the visualization of the mucosal surface of the scoped organ. A small area of inflamed, red, and possibly elevated mucosa are signs of possible fistulous tract opening. Unless the fistula is very wide, it is usually difficult to visualize its lumen endoscopically. Endoscopy can provide further information about the underlying disease such as malignancy or Crohn’s disease. Fistulae might be an incidental finding of endoscopy that is performed for other reasons. In this situation, further investigations are required.

Treatment/Management

The appropriate management of small bowel fistulae is based on four prongs: (1) optimizing nutritional status, (2) delineating fistulous tract anatomy, (3) skincare, and (4) managing the underlying disease [34]. A small bowel fistula is, as discussed above, a complication of an underlying disease, procedure, foreign body, or injury. Treatment will, therefore, include the fistula itself and the underlying pathology when treatable. Therefore, confirming the fistula etiology is essential and should be done before planning treatment. A good clinical practice is to treat with the least aggressive and safest approach. This generally includes a trial of non-operative management aimed at addressing nutrition, skincare, and the underlying disease.

Non-operative Approach

Medical treatment of the symptoms and possible complications of the small bowel fistula can be used as sufficient first-line treatment in selected patients, particularly in low output fistulas. This approach can also be considered in high-risk patients with severe underlying disease. The associated complication rate from this approach is found to be low in recent studies [35-36]. 

The basic principle of non-operative treatment is to control the fistula in the acute setting and prevent further complications such as sepsis, skin complications, or dehydration. Medical treatment includes issues related to skincare and protection, optimizing nutrition, antibiotics, maximizing the medical treatment of the underlying disease such as in Crohn's disease or diverticulitis, and support of the patient’s general nutritional status. Total parenteral nutrition (TPN) with complete bowel rest is sometimes advocated in high-output fistula to facilitate healing and decreasing output [34].

Endoscopic Therapies

These therapies are gaining wider application with the advancement of endoscopic therapy techniques. This is managed via an endoluminal approach with covered stents, sealants, clips, and plugs [37-38].

Operative Approach

Operating for newly diagnosed fistulas should ideally be delayed for at least three months given the degree of inflammation and the risk of creating further injuries. The basic principle of the surgical approach is to excise the involved segment of the bowel and the fistula. After the diagnosis of the fistula and confirmation of the underlying disease with sufficient characterization, surgical treatment can be planned accordingly. Limited conservative excision of the involved intestinal segment and the fistula is recommended in operative cases of diverticular disease, Crohn's disease, and other reversible inflammatory diseases. More radical and oncologic excision is recommended in surgically treatable malignancy. Special considerations with specific fistulae should be taken.

Enhancing healthcare team outcomes

The management of enterovesical fistulae is potentially challenging. It requires assessment and planning by a multidisciplinary team. Suspected fistula patients should be appropriately referred and investigated. Proper planning and the involvement of the required services, such as a colorectal surgeon, dietitian, colostomy nurse, general surgeon, and gastroenterologist, are essential. Other specialties, such as oncology, radiology, wound therapy, nutritionist, and nursing, are usually also needed when caring for patients with a small bowel fistula.

## Conclusions

A small bowel fistula is a complex clinical disease, with surgical intervention being the most common cause in developed countries. A non-operative approach should be trialed before an operative approach is considered. A multidisciplinary team approach should be started early in the disease process with the goal of nutritional support, skin protection and delineation of anatomy. The operative approach provides definitive management and is usually attempted after the failure of non-operative management.
